# Multi-Exposure and Clustering of Adverse Childhood Experiences, Socioeconomic Differences and Psychotropic Medication in Young Adults

**DOI:** 10.1371/journal.pone.0053551

**Published:** 2013-01-16

**Authors:** Emma Björkenstam, Anders Hjern, Ellenor Mittendorfer-Rutz, Bo Vinnerljung, Johan Hallqvist, Rickard Ljung

**Affiliations:** 1 Department of Public Health Sciences, Division of Social Medicine, Karolinska Institutet, Stockholm, Sweden; 2 Department of Statistics, Monitoring and Evaluation, National Board of Health and Welfare, Stockholm, Sweden; 3 Centre for Health Equity Studies (CHESS), Stockholm University/Karolinska Institutet, Stockholm, Sweden; 4 Department of Clinical Neuroscience, Division of Insurance Medicine, Karolinska Institutet, Stockholm, Sweden; 5 Department of Social Work, Stockholm University, Stockholm, Sweden; 6 Department of Public Health Sciences, Division of Public Health Epidemiology, Karolinska Institutet, Stockholm, Sweden; 7 Department of Public Health and Caring Sciences, Uppsala University, Uppsala, Sweden; 8 Upper Gastrointestinal Research, Department of Molecular Medicine and Surgery, Karolinska Institutet, Stockholm, Sweden; Catholic University of Sacred Heart of Rome, Italy

## Abstract

**Purpose:**

Stressful childhood experiences have negative long-term health consequences. The present study examines the association between adverse childhood experiences, socioeconomic position, and risk of psychotropic medication in young adulthood.

**Methods:**

This register-based cohort study comprises the birth cohorts between 1985 and 1988 in Sweden. We followed 362 663 individuals for use of psychotropic medication from January 2006 until December 2008. Adverse childhood experiences were severe criminality among parents, parental alcohol or drug abuse, social assistance recipiency, parental separation or single household, child welfare intervention before the age of 12, mentally ill or suicidal parents, familial death, and number of changes in place of residency. Estimates of risk of psychotropic medication were calculated as odds ratio (OR) with 95% confidence intervals (CIs) using logistic regression analysis.

**Results:**

Adverse childhood experiences were associated with increased risks of psychotropic medication. The OR for more than three adverse childhood experiences and risk of psychotropic medication was for women 2.4 (95% CI 2.3–2.5) and for men 3.1 (95% CI 2.9–3.2). The risk of psychotropic medication increased with a higher rate of adverse childhood experiences, a relationship similar in all socioeconomic groups.

**Conclusions:**

Accumulation of adverse childhood experiences increases the risk of psychotropic medication in young adults. Parental educational level is of less importance when adjusting for adverse childhood experiences. The higher risk for future mental health problems among children from lower socioeconomic groups, compared to peers from more advantaged backgrounds, seems to be linked to a higher rate of exposure to adverse childhood experiences.

## Introduction

Studies on adverse childhood experiences (ACEs) strongly indicate that stressful childhood experiences (e.g. abuse, neglect, growing up with parental substance abuse) can have negative long term health and social consequences [1]–[4]. Evidence from neurobiology and epidemiology suggests that early life stress causes enduring brain dysfunction that affects health and quality of life throughout the lifespan [1].

Accumulation of risks over the life span has been suggested as one etiologic pathway in a life course approach to persistent mental health problems. Adverse childhood experiences tend to occur in clusters, rather than as single experiences [4], [5]. Several studies have shown that clustered adverse experiences during the formative years have a strongly graded relationship to several mental health problems, from adolescence to adulthood [4], [6]–[8]. This is congruent with the allostatic stress theory, which suggests that the neurobiological stress management systems can be permanently altered by cumulative/chronic stress in childhood [9], [10].

Socioeconomic variation in the risk of mental disorder may be explained by differential clustering of risk factors by socioeconomic position. Between-group clustering refers to clustering of risk factors in different socioeconomic groups, and within-group clustering describes clustering of risk factors within a socioeconomic group [11]. Risk factor clustering may be distributed differentially between as well as within socioeconomic groups [11], [12]. Low socioeconomic position in childhood increases the risk of mental disorder in adulthood [13]–[15]. Also, low socioeconomic position in childhood is associated with exposure to a range of risk factors for both somatic and mental disorder [16], [17].

Psychotropic drugs are commonly used in the adult population [18]. Their main use is to treat mental disorder and mental health problems, though there are other indications as well like selective serotonin reuptake inhibitors (SSRI) for the treatment of pain or premenstrual syndrome [19]. Few studies have assessed the relationship of adverse childhood experiences and the use of psychotropic drugs in adulthood [6]. With the aim of clarifying the association between socioeconomic position, adverse childhood experiences, and risk of psychotropic medication in young adulthood, we conducted a large nationwide register-based cohort study with long and complete follow-up. Adverse childhood experiences were measured as severe criminality among parents, parental alcohol or drug abuse, social assistance recipiency, parental separation or single household, child welfare intervention before the age of 12 mentally ill or suicidal parents, familial death, and number of changes in place of residency. Secondary aims were to explore whether the risk of psychotropic medication increases with increasing number of adverse childhood experiences, and whether adverse childhood experiences cluster between and within socioeconomic groups.

## Methods

### Study population

The study population was defined as all individuals born in Sweden between 1985 and 1988 with at least one parent born in Sweden (n = 382 154) and recorded in the Medical Birth Register. This register was founded in 1973 and includes data on practically all deliveries in Sweden [20].

Individuals who had ever migrated (n = 15 012), been adopted (n = 227), diagnosed with mental retardation during the ages 0–17 (n = 392), and those who died before the 31^st^ of December 2008 (n = 3 954) were excluded. After excluding these 19 491 individuals our final cohort comprised 175 626 women and 187 037 men. Individuals were followed for use of psychotropic medication from the 1^st^ of January 2006 until the 31^st^ of December 2008. Thus the prevalence of psychotropic medication was measured from the age of 18 to 20 for those born in 1988, and from the age of 21 to 23 for those born in 1985.

Swedish national registers make it possible to study the entire Swedish population due to individual record linkage between different registers. The unique personal identity number assigned to each Swedish citizen or permanent resident was used to link information from several population-based registers [21]. The Crime Register contains information on all court convictions in Sweden from the age of 15 years. The National Patient Register includes all individuals admitted to any psychiatric or general hospital since 1987. The Total Enumeration Income Survey contains data on the income on all Swedish residents. The Swedish Register of Children and Young Persons Subjected to Child Welfare Measures has records on out-of-home care, foster family and residential care. The Swedish Population and Housing Census was a mandatory nationwide census conducted every 5 years between 1960 and 1990. The Causes of Death Register contains information on all deceased Swedish residents since 1952. The Swedish Register of Education comprises records from most completed educational programs offered in Sweden. Finally, the Prescribed Drug Register contains patient identities for all dispensed prescribed drugs to the entire Swedish population since July 2005 [22].

### Definitions of adverse childhood experiences

In total, we studied eight adverse childhood experiences, experiences that alone have been shown to be risk factors for mental health problems. All adverse childhood experiences were measured somewhere between birth and age 14.

#### Severe criminality among parents

Children growing up with criminal parents have increased risk for mental health problems [23]. This adverse childhood experience, measured from birth until age 14, was defined as parents who received custodial or noncustodial sentences or individuals transferred to forensic hospitals.

#### Familial death

Death of a family member is regarded a traumatic life event that increases stress levels in children [24], [25]. We defined familial death as death of either parent or sibling. This adverse childhood experience was measured from birth until age 14.

#### Parental alcohol or drug abuse

This is a commonly used measure of adverse childhood experience [6]. Parents hospitalized with a main diagnosis for alcohol and/or narcotic-related substance abuse as defined by the International Classification of Disease (ICD): ICD-9: 291, 303, 305A, 357F, 425F, 535D, 571A, 571B, 571C, 571D, 292, 304, 648D, 655F, 969G, 969H, and 965A; ICD-10: E24.4, F10, G31.2, G62.1, G72.1, I42.6, K29.2, K70, K85.2, K86, O35.4, T51, Z50.2, Z71.4, Z72.1, F11-16, F18-19, O35.5, P04.4, T40, T43.6, Z50.3, Z71.5, and Z72.2, or parents who had received an alcohol or narcotic-related drug conviction. This adverse childhood experience was measured from 1987 and onwards.

#### Parents with mental health problems

Children of mentally ill parents are at greater risk of mental health problems [26]. Parents hospitalized with a main diagnosis of mental disorder as defined by ICD-9: 290–319 (substance abuse excluded); ICD-10 and F00-F98 (substance abuse excluded) respectively. Suicidal parents included parents hospitalized with a diagnosis for intentional self-harm (including events of undetermined intent) as defined by ICD-9:E950-E959, E980E-989; ICD-10: X60-X84, Y10-Y34. This adverse childhood experience was measured from 1987 and onwards.

#### Social assistance recipiency

Children in families receiving long-term social assistance face less satisfactory health [27]. To fulfill the criterion of “receiving social assistance”, at least one parent must receive social assistance at least one year (measured when the child was between 5 and 14 years old), where more than 50 percent of the yearly income constituted social assistance.

#### Parental separation or single household

Growing up in a single-parent family has disadvantages for the health of the child [28]. Single household was measured when the child was between 5 and 14 years old.

#### Child Welfare Intervention

Experience of interventions before age 12, a well-known risk factor for mental health problems [29], [30], was defined as out-of-home care or provision of respite care.

#### Number of changes in place of residency

Frequent changes of residence during childhood are associated with an increased risk of mental health problems [31]. We defined this adverse childhood experience as two or more changes when the child was between the ages 6 and 14, i.e. the schooling years.

To assess the cumulative effect of multiple adverse childhood experiences, we summed the total number of experiences into the following categories: zero, one, two, three or more adverse childhood experiences.

### Measures of socioeconomic position

Highest attained parental education level was measured when the child was 15 years (between 2000 and 2003). For households with two adults, we chose the highest educational level to characterize the household. Educational level was classified into five categories: 1: nine years of compulsory school, 2: 10–12 years of education (equivalent to senior high school), 3: 13–14 years of education (i.e. university education less than 3 years), 4: ≥15 years of education, 5: Missing.

### Psychotropic medication

Treatment with psychotropic medication was used as a proxy for mental disorder. Pharmaceuticals were grouped according to Anatomical Therapeutic Chemical Classification System (ATC): neuroleptics (N05A), anxiolytics and sedatives (N05B–N05C), antidepressants (N06A). An individual was considered to have received drug treatment if at least one prescription for psychotropic medication was dispensed between 2006 and 2008.

We created a variable “place of residence” to adjust for potential confounding by population density. The variable was classified into three categories: small (rural municipalities), intermediate (>90 000 inhabitants), and big city area (the three large city areas).

### Statistical analysis

Observed and expected prevalence for each adverse childhood experience was calculated for the whole cohort as well as for each stratum of parental socioeconomic position. We derived expected frequencies of co-occurrence of adverse childhood experiences for all combinations of adverse childhood experiences by combining probabilities, assuming a binomial distribution and independence between them. For the between-group clustering analyses the prevalence in the total population was used. For within group clustering analyses, prevalence in each specific stratum was used. Based on the assumption that exposure to the different adverse childhood experiences was independent of the sex of the child, women and men were analyzed in one group. Clustering is indicated when individuals are more likely to have no or many adverse childhood experiences and thus are less likely to have a single or few adverse childhood experiences than would be expected if the distribution of adverse childhood experiences was independent (thus observed-to-expected ratio greater than one indicates clustering).

The psychotropic medication groups were defined as binary variables. Logistic regression analyses were used to statistically evaluate the association between adverse childhood experience and psychotropic medication utilization. To further study the interaction between parental education and number of adverse childhood experiences in the risk of psychotropic medication, synergy index (SI) was calculated. SI measures the interaction, expressed as the ratio of the relative excess risk for the combined effect of the risk factors and the sum of the relative excess risks for each separate effect of the two risk factors. An SI greater than 1 indicates that the absolute excess risk for those exposed to both risk factors is greater than the sum of the absolute excess risks for those exposed to each separate risk factor [32].

For all statistical analyses, SAS v. 9.2 (SAS Institute Inc. Cary, NC, USA) was used.

### Ethics statement

The study population was based on linkage of several public national registers. Ethical vetting is always required when using register data in Sweden. The ethical vetting is performed by regional ethical review boards and the risk appraisal associated with the Law on Public Disclosure and Secrecy is done by data owners. The ethical review boards can however waive the requirement to consult the data subjects (or in case of minors/children the next of kin, careers or guardians) directly to obtain their informed consent, and will often do so if the research is supported by the ethical review board and the data has already been collected in some other context. According to these standards in Sweden this project has been evaluated and approved by the Regional Ethical Review Board of Karolinska Institutet, Stockholm, Sweden.

## Results

Of the 362 663 individuals, 40 percent had been exposed to at least one adverse childhood experience during childhood and nearly 20 percent had two or more adverse childhood experiences. The cohort was equally distributed between women and men (48% women and 52% men) across all educational groups. A majority of all individuals had parents with 10–12 years of education (51%). Only five % had parents with a maximum of nine years of education. The prevalence of adverse childhood experiences is shown in table 1. Exposure to adverse childhood experiences was more common among individuals whose parents had low socioeconomic position. Nearly 35% of the individuals had experienced at least one parental separation or were living in a single household. Around four % had moved more than two times during the schooling years. More than 50% of all individuals had parents with 10–12 years of education. Only five % had parents with a maximum of nine years of education.

**Table 1:** Prevalence of adverse childhood experiences by parental educational level.

	Parental education				Total**
	≥15 years of education	13–14 years of education	10–12 years of education	9 years of education	
N	87 218	71 262	184 435	19 582	362 663
Women	42 236 (48.4%)	34 406 (48.3%)	89 295 (49.1%)	9 608 (49.1%)	175 626 (48.4%)
Men	44 982 (51.6%)	36 856 (51.7%)	95 140 (50.9%)	9 974 (50.9%)	187 037 (51.6%)
**Adverse Childhood Experiences**					
Severe crime parents	1 090 (1.2%)	1 386 (1.9%)	9 205 (5.0%)	1 736 (8.9%)	13 444 (3.7%)
Parental alcohol and/or drug abuser	2 822 (3.2%)	3 126 (4.4%)	17 345 (9.4%)	3 016 (15.4%)	26 366 (7.3%)
Parental separation and/or single household	22 259 (25.5%)	21 013 (29.5%)	72 342 (39.2%)	9 417 (48.1%)	125 168 (34.5%)
Household receiving social assistance	5 332 (6.1%)	7 240 (10.2%)	40 786 (22.1%)	6 944 (35.5%)	60 983 (16.8%)
Child welfare intervention before the age of 12	875 (1.0%)	971 (1.4%)	7 492 (4.1%)	1 877 (9.6%)	11 312 (3.1%)
Mentally ill or suicidal parent	3 512 (4.0%)	3 220 (4.5%)	13 009 (7.1%)	2 195 (11.2%)	22 001 (6.1%)
Familial death	1 977 (2.3%)	1 690 (2.4%)	6 641 (3.6%)	2 119 (10.8%)	12 568 (3.5%)
Two or more changes in place of residence	2 495 (2.9%)	1 901 (2.7%)	7 706 (4.2%)	1 251 (6.4%)	13 380 (3.7%)
**Number of adverse childhood experiences**					
0	60 205 (69.0%)	46 407 (65.1%)	97 913 (53.1%)	7 963 (40.7%)	212 491 (58.6%)
1	18 653 (21.4%)	15 886 (22.3%)	42 592 (23.1%)	4 015 (20.5%)	81 152 (22.4%)
2	5 631 (6.5%)	5 767 (8.1%)	23 071 (12.5%)	3 339 (17.1%)	37 843 (10.4%)
3+	2 729 (3.1%)	3 202 (4.5%)	20 859 (11.3%)	4 265 (21.8%)	31 177 (8.6%)

Observed (O) and expected (E) prevalence (%) of exposure to increasing number of adverse childhood experiences and observed-to-expected ratios by parental socioeconomic position are presented in table 2. The between-group analysis shows a higher-than-expected number of individuals who had no adverse childhood experiences for those whose parents had high socioeconomic position, compared to the total population (O = 69.2% E = 60.6%, O:E = 1.7 (95% CI 1.3–2.1)). Higher-than-expected O:Es were observed in participants who had three or more adverse childhood experiences whose parents had 10–12 years of education, and 9 years of education, compared to the total population (O:E = 4.4 (95% CI 2.2–7.8) and O:E = 8.4 (95% CI 5.3–12.8) respectively). Within all socioeconomic groups, the adverse childhood experiences were clustered with a greater-than-expected number of individuals who had no adverse childhood experiences, a lower-than-expected number of individuals who had one adverse childhood experience, and a greater-than-expected number of individuals who had three or more adverse childhood experiences. Similar results were obtained when the analyses were stratified by the sex of the child (data not shown).

**Table 2:** Observed (O) and expected (E) prevalence (%) of exposure to increasing number of adverse childhood experiences and observed-to-expected ratio by parental educational level.

Number of adverse childhood experiences	ALL			Parental education															
				≥15 years of education				13–14 years of education				10–12 years of education				9 years of education			
	O	E	O:E	O	E	Between-group O:E	Within-group O:E	O	E	Between-group O:E	Within-group O:E	O	E	Between-group O:E	Within-group O:E	O	E	Between-group O:E	Within-group O:E
0	58.6	42.5	1.4 (1.1–1.8)	69.2	60.6	**1.7 (1.3–2.1)**	1.2 (0.9–1.5)	65.4	54.0	**1.6 (1.2–2.0)**	1.2 (0.9–1.6)	53.5	34.7	**1.3 (1.0–1.7)**	1.6 (1.2–2.1)	41.0	18.3	**1.0 (0.7–1.3)**	2.3 (1.7–3.2)
1	21.8	41.8	0.5 (0.3–0.8)	21.4	33.5	**0.5 (0.3–0.8)**	0.6 (0.4–1.0)	22.3	37.5	**0.5 (0.3–0.8)**	0.6 (0.4–0.9)	23.3	43.4	**0.6 (0.3–0.8)**	0.5 (0.3–0.8)	20.8	38.7	**0.5 (0.3–0.8)**	0.5 (0.3–0.8)
2	10.8	13.5	0.8 (0.4–1.4)	6.4	5.4	**0.4 (0.2–0.9)**	1.1 (0.4–2.4)	8.1	7.8	**0.6 (0.2–1.1)**	1.0 (0.4–1.9)	12.8	18.1	**0.9 (0.5–1.5)**	0.7 (0.3–1.1)	17.6	29.7	**1.2 (0.9–1.9)**	0.6 (0.3–0.9)
3+	8.7	2.2	3.9 (1.8–7.9)	2.9	0.4	**1.2 (0.3–3.5)**	6.4 (1.3–18.3)	4.1	0.8	**1.7 (0.5–4.2)**	5.1 (1.5–12.4)	10.4	3.8	**4.4 (2.2–7.8)**	2.6 (1.3–4.6)	20.7	13.4	**8.4 (5.3–12.8)**	1.5 (0.9–2.2)

P values were the same (p<0.001) across all educational levels. P values were on the basis of χ^2^ test with 3 degrees of freedom testing the null hypothesis of no differences in the observed and expected frequencies across all number of ACE categories. Given the prevalence of the risk factors within each group and on the basis of the assumption that all of the ACEs were independent of each other. The between-group comparison used the expected value from the total number of individuals as comparison.

Of the women, 23 395 women and 13 407 men received psychotropic medication during the follow-up period. The proportion of women on psychotropic medication was nearly twice as high, compared to men (table 3). As the number of adverse childhood experiences increased, rates of at least one filled prescription of psychotropic medication increased in a graded fashion. This relationship was similar in all socioeconomic groups. Nearly one in four of the women who had experienced more than three adverse childhood experiences received psychotropic medication, compared to approximately one in seven of the men experiencing more than three adverse childhood experiences.

**Table 3:** Rates per 100 of at least one retrieved recipe with psychotropic medication during 2006–2008 by parental educational level and exposure to increasing number of adverse childhood experiences.

	Women					Men				
Number of adverse childhood experiences	Parental education				All	Parental education				All
	>15 years	13–14 years	10–12 years	9 years		>15 years	13–14 years	10–12 years	9 years	
0	10.9 (10.5–11.3)	10.3 (9.9–10.6)	10.8 (10.6–11.1)	11.7 (10.7–12.7)	10.8 (10.6–11.0)	5.8 (5.5–6.0)	5.0 (4.7–5.3)	5.2 (5.0–5.4)	5.4 (4.7–6.1)	5.3 (5.2–5.4)
1	14.6 (13.9–15.3)	13.5 (12.8–14.3)	14.9 (14.4–15.4)	15.4 (3.8–17.0)	14.6 (14.2–14.9)	8.0 (7.4–8.5)	7.5 (6.9–8.1)	7.7 (7.3–8.0)	8.2 (7.0–9.4)	7.7 (7.5–8.0)
2	16.7 (15.3–18.1)	18.0 (16.6–19.5)	17.2 (16.5–17.9)	17.6 (15.7–19.4)	17.3 (16.7–17.8)	10.1 (9.0–11.2)	9.7 (8.6–10.7)	10.7 (10.1–11.3)	9.4 (8.0–10.8)	10.3 (9.9–10.8)
3+	20.4 (18.2–22.6)	22.4 (20.3–24.5)	22.4 (21.6–23.2)	24.9 (23.1–26.8)	22.6 (21.9–23.2)	15.2 (13.3–17.0)	15.5 (13.8–17.3)	14.3 (13.6–14.9)	15.6 (14.0–17.1)	14.6 (14.1–15.2)

The OR indicated an increased risk of all types of psychotropic medications with increasing number of adverse childhood, in both women and men (table 4 and table 5) The OR for more than 3 adverse childhood experiences and risk of antidepressants, neuroleptics, anxiolytics and sedatives, was for women 2.4 (95% CI 2.3–2.5), 3.1 (95% CI 2.8–3.5), and 2.5 (95% CI 2.4–2.7) respectively. Corresponding ORs for men were 3.1 (95% CI 2.9–3.3), 4.3 (95% CI 3.8–4.9), and 3.1 (95% CI 2.9–3.0). Among women who had more than three adverse childhood experiences, we found slightly lower risks of neuroleptics, for individuals whose parents had 15 years or more of education, compared with the other educational groups, which was not seen among men.

**Table 4:** Risk of psychotropic medication 2006–2008 (OR, 95% confidence intervals) by parental education and exposure to increasing number of adverse childhood experiences (Women).

	Number of adverse childhood experiences				
	0+	0	1	2	3+
**Any psychotropic medication**					
All		1 (REF)	1.4 (1.4–1.5)	1.8 (1.7–1.8)	2.4 (2.3–2.5)
≥15 years	1 (REF)	1 (REF)	1.4 (1.3–1.5)	1.6 (1.5–1.8)	2.1 (1.8–2.4)
13–14 years	0.9 (0.9–1.0)	0.9 (0.9–1.0)	1.3 (1.2–1.4)	1.8 (1.6–2.0)	2.4 (2.1–2.7)
10–12 years	1.1 (1.1–1.2)	1.0 (1.0–1.0)	1.5 (1.4–1.5)	1.7 (1.6–1.8)	2.4 (2.2–2.5)
9 years	1.4 (1.3–1.5)	1.1 (1.0–1.2)	1.4 (1.3–1.6)	1.7 (1.5–2.0)	2.7 (2.5–3.0)
**Antidepressants** *					
All		1 (REF)	1.4 (1.4–1.5)	1.7 (1.7–1.8)	2.4 (2.3–2.5)
≥15 years	1 (REF)	1 (REF)	1.4 (1.3–1.5)	1.5 (1.4–1.7)	2.1 (1.8–2.4)
13–14 years	1.0 (0.9–1.0)	0.9 (0.9–1.0)	1.3 (1.2–1.4)	1.9 (1.7–2.1)	2.4 (2.0–2.7)
10–12 years	1.1 (1.0–1.1)	0.9 (0.9–1.0)	1.3 (1.3–1.4)	1.6 (1.5–1.7)	2.2 (2.1–2.4)
9 years	1.3 (1.2–1.4)	1.0 (0.9–1.1)	1.4 (1.2–1.6)	1.7 (1.5–2.0)	2.6 (2.3–2.9)
**Neuroleptics** *					
All		1 (REF)	1.4 (1.4–1.5)	1.8 (1.7–1.8)	2.4 (2.3–2.5)
≥15 years	1 (REF)	1 (REF)	1.4 (1.3–1.5)	1.6 (1.5–1.8)	2.1 (1.8–2.4)
13–14 years	0.9 (0.9–1.0)	0.9 (0.9–1.0)	1.3 (1.2–1.4)	1.8 (1.6–2.0)	2.4 (2.1–2.7)
10–12 years	1.1 (1.1–1.2)	1.0 (1.0–1.0)	1.5 (1.4–1.5)	1.7 (1.6–1.8)	2.4 (2.2–2.5)
9 years	1.4 (1.3–1.5)	1.1 (1.0–1.2)	1.4 (1.3–1.6)	1.7 (1.5–2.0)	2.7 (2.5–3.0)
**Anxiolytics and sedatives** *					
All		1 (REF)	1.4 (1.4–1.5)	1.7 (1.7–1.8)	2.4 (2.3–2.5)
≥15 years	1 (REF)	1 (REF)	1.4 (1.3–1.5)	1.5 (1.4–1.7)	2.1 (1.8–2.4)
13–14 years	1.0 (0.9–1.0)	0.9 (0.9–1.0)	1.3 (1.2–1.4)	1.9 (1.7–2.1)	2.4 (2.0–2.7)
10–12 years	1.1 (1.0–1.1)	0.9 (0.9–1.0)	1.3 (1.3–1.4)	1.6 (1.5–1.7)	2.2 (2.1–2.4)
9 years	1.3 (1.2–1.4)	1.0 (0.9–1.1)	1.4 (1.2–1.6)	1.7 (1.5–2.0)	2.6 (2.3–2.9)

Adjusted for birth year and place of residence.

* Antidepressants: ATC N06A; Neuroleptics: ATC N05A; Anxiolytics and sedatives: ATC N05B-C.

**Table 5:** Risk of psychotropic medication (OR, 95% confidence intervals) by parental education and exposure to increasing number of adverse childhood experiences. (Men).

	Number of adverse childhood experiences				
	0+	0	1	2	3+
**Any psychotropic medication**					
All		1 (REF)	1.5 (1.4–1.6)	2.1 (2.0–2.2)	3.1 (2.9–3.2)
≥15 years	1 (REF)	1 (REF)	1.4 (1.3–1.5)	1.8 (1.6–2.1)	2.9 (2.5–3.4)
13–14 years	1.0 (0.9–1.0)	0.9 (0.8–0.9)	1.3 (1.2–1.5)	1.8 (1.6–2.0)	3.0 (2.6–3.5)
10–12 years	1.1 (1.1–1.2)	0.9 (0.8–0.9)	1.4 (1.3–1.5)	2.0 (1.8–2.1)	2.7 (2.5–2.9)
9 years	1.3 (1.2–1.4)	0.9 (0.8–1.0)	1.4 (1.2–1.7)	1.7 (1.4–2.0)	3.0 (2.7–3.4)
**Antidepressants** *					
All		1 (REF)	1.5 (1.4–1.6)	2.1 (2.0–2.2)	3.1 (2.9–3.3)
≥15 years	1 (REF)	1 (REF)	1.4 (1.2–1.5)	1.9 (1.6–2.2)	3.2 (2.7–3.8)
13–14 years	0.9 (0.8–1.0)	0.8 (0.7–0.9)	1.3 (1.1–1.4)	1.8 (1.5–2.1)	3.0 (2.6–3.6)
10–12 years	1.0 (1.0–1.1)	0.8 (0.8–0.9)	1.3 (1.2–1.4)	1.8 (1.7–2.0)	2.6 (2.4–2.8)
9 years	1.2 (1.1–1.3)	0.8 (0.7–1.0)	1.3 (1.1–1.6)	1.5 (1.2–1.8)	2.6 (2.2–3.1)
**Neuroleptics** *					
All		1 (REF)	1.7 (1.5–2.0)	2.6 (2.3–3.0)	4.3 (3.8–4.9)
≥15 years	1 (REF)	1 (REF)	1.7 (1.4–2.2)	2.5 (1.8–3.4)	4.4 (3.1–6.2)
13–14 years	1.0 (0.9–1.2)	0.9 (0.7–1.1)	1.6 (1.2–2.0)	2.3 (1.7–3.2)	4.9 (3.5–6.7)
10–12 years	1.1 (1.0–1.2)	0.8 (0.7–1.0)	1.5 (1.3–1.9)	2.4 (1.9–2.9)	3.6 (3.0–4.4)
9 years	1.3 (1.1–1.7)	0.8 (0.5–1.3)	1.4 (0.8–2.2)	1.8 (1.1–2.8)	4.3 (3.2–5.8
**Anxiolytics and sedatives** *					
All		1 (REF)	1.5 (1.4–1.6)	2.1 (2.0–2.2)	3.1 (2.9–3.0)
≥15 years	1 (REF)	1 (REF)	1.5 (1.3–1.6)	1.8 (1.5–2.1)	2.8 (2.3–3.4)
13–14 years	1.0 (0.9–1.1)	0.9 (0.8–1.0)	1.4 (1.3–1.6)	2.0 (1.7–2.3)	3.0 (2.5–3.6)
10–12 years	1.2 (1.1–1.3)	1.0 (0.9–1.1)	1.5 (1.4–1.6)	2.2 (2.0–2.4)	3.0 (2.8–3.3)
9 years	1.5 (1.3–1.6)	1.0 (0.9–1.2)	1.5 (1.2–1.8)	1.9 (1.6–2.3)	3.4 (2.9–3.9)

Adjusted for birth year and place of residence.

* Antidepressants: ATC N06A; Neuroleptics: ATC N05A; Anxiolytics and sedatives: ATC N05B-C.

In the SI analysis (data not shown), we merged all individuals with any adverse childhood experience to one group. The four groups for parental education were reduced to two groups: 9–12 years of education and >12 years of education. The risk of psychotropic medication for those exposed to at least one adverse childhood experience *and* having parents with 9–12 years of education was higher than would be expected from the additive effect of the two exposures (SI: 1.4 (95% CI 1.2–1.5) when adjusting for birth year and sex.

The highest OR for both sexes is observed for the event “child welfare intervention before the age of 12”, and lowest OR is seen for “familial death (table S1).

As parental mental health problems can influence offspring mental health, we conducted a sub analysis in which we excluded “parental mental health problems” from the list of adverse childhood experiences (data not shown). Results from this analysis did not differ significantly from the main analysis.

## Discussion

Our study of 362 663 young adults shows that adverse childhood experiences were associated with increased risks of psychotropic medication in young adulthood. Adverse childhood experiences were more common in individuals whose parents had low socioeconomic position. The risk of psychotropic medication increased with a higher rate of adverse childhood experiences. Socioeconomic position did not modify the association between number of adverse childhood experiences and risk of psychotropic medication.

The strengths of the study include the population-based design, using national registers with high completeness and validity [22], [33], [34]. Earlier studies on adverse childhood experiences as risk factors for adverse health in adulthood have often been retrospective studies based on self-reporting information with risk for recall bias. In this study we were able to study a large cohort using prospective data without any recall bias, as both the exposure and outcome measures used register data.

There are, however, several weaknesses. We used treatment with psychotropic medication as a proxy for mental health problems. Thus, we only capture mental health problems that are in part treated with psychotropic drugs. A study examining the diagnoses for which psychotropic medication are being prescribed in the US showed that, even though most prescribing of psychotropic medication is for psychiatric conditions, these drugs also have other indications than psychiatric disorders [19]. That study also showed that the types of psychiatric conditions being treated are quite varied, which highlights the complexity and challenges of psychiatric diagnosis and treatment. A European study on psychotropic drug utilization showed that 10% of individuals without a diagnosis of any mental disorder also reported using a psychotropic drug [35]. Hence, individuals treated with psychotropic drugs for other indications are classified as having mental health problems. However, if our studied exposures are less associated with these non-psychiatric indications this misclassification of disease will lead to an underestimation of the association between childhood adverse events and psychotropic drug use.

Relying solely on register data, there are important adverse childhood experiences (e.g. factors such as malnutrition and serious peer victimization) that we were not able to study. Also, hospital admissions account for only a small part of all psychiatric interventions in Sweden, as most services are delivered in out-patient care. It was first in 2001 that specialized out-patient care was available in the National Patient Register, thus only in-patient data were used in this study. Hence, we only capture the most severe forms of mental health problems and substance abuse among parents requiring in-patient care. Another important limitation in this study is the use of parental psychiatric morbidity as an exposure of adverse childhood, since one can expect that this exposure is not only an environmental risk factor, but also a genetic risk factor, for psychotropic medication.

It should also be kept in mind that rates of adverse childhood experiences vary considerably between societies, depending on affluence, child rearing patterns and welfare system. A recent comparison of rates of child maltreatment between six high income countries, for instance, showed that Sweden had the lowest rates [36], and Sweden is also known to have a comparably generous welfare system for families with children. Thus, one might expect that the importance of adverse childhood experiences for the health of young adults is even greater in many other high-income countries.

Our results show an increased risk of psychotropic medication as the number of adverse childhood experiences increase. Adverse childhood experiences tend to occur in cluster and they are often associated with each other. A survey study on 8 629 adults showed that the presence of one adverse childhood experience significantly increased the prevalence of having additional experiences [5]. This pattern could also be seen in this study, stressing the importance of controlling for exposure to other adverse childhood experiences when studying the independent consequence of exposure to a specific adverse childhood experience. Thus, these experiences should not be assumed to be isolated events in children's lives. Our cluster analysis showed a lower than expected number of participants who had one adverse childhood experience. We found a greater than expected prevalence of null- and multi exposed individuals in the whole population as well as in the different educational groups. However, our results showed that, for those with more than three adverse childhood experiences, between-clustering was more common among those whose parents had lower educational level. Within group clustering was similar in all educational groups. A cross sectional study on clustering of risk factors by social class in childhood and adult life showed that co-occurrence of three or four risk factors was greater among more disadvantaged groups [37]. Within each of these different groups of social class, risk factors co-occurred more than would be expected, indicating within-group clustering. Despite this fact, clustering of risk factors occurs within all social classes showing no evidence that the extent of within-group clustering is greater among those in lower socioeconomic groups [11], [12], [37], [38].

There was no difference between the sexes in the association between adverse childhood experiences and risk of psychotropic medication. However, women had nearly twice as high rates of dispensed prescriptions for psychotropic medication compared to men, as also shown in other settings [35], [39].

Every adverse childhood experience on its own increased the likelihood of psychotropic medication regardless of parental educational level (table S1). Several of these adverse childhood experiences reflect the family environment in childhood, and previous studies have highlighted the strong influence of the family environment on later mental health [3], [40]. A study using similar adverse childhood experiences as we did on allostatic load showed that children who face more cumulative risk have greater psychological distress [9].

The dose-response relationship between the burden of adverse childhood experiences and the risk of psychotropic medication was true for all groups of psychotropics. This indicates that adverse childhood experiences are associated with a broad range of negative psychological consequences.

Among individuals with no adverse childhood experiences, there were no differences in the risk of psychotropic medication due to parental educational level, showing that number of adverse childhood experiences was an important factor, not parental educational level. However as it is more common for adverse childhood experiences to be accumulated among individuals in lower socioeconomic groups, an increased risk for psychotropic medication was observed in individuals in lower parental educational groups. Hence, when taking number of adverse childhood experiences into account the risk of psychotropic medication was the same for all educational groups. This could reflect that our measures of adverse childhood experiences capture a large part of the most important risk factors for future psychotropic medication. However, it could also be the effect of disparities in access to and utilization of the health care system where individuals with lower parental education do not get access to adequate psychotropic medication to the same extent as those with higher parental educational level.

A Swedish study on education and drug use showed that those with low education generally are at higher risk of using drugs but, for some exclusive drugs, people with higher education had a higher consumption than expected [41].

Several previous studies have pointed out a strong link between severe adverse childhood experiences, in particular child abuse and neglect, and development of psychiatric problems [1], [18]. One explanation to how these problems come about is that early maltreatment has enduring negative effects on brain development. The adverse childhood experiences in this study are indicators of less severe everyday life stressors. Our study thus seems to support the allostatic stress theory in its claims that accumulation of stress, not only extreme deprivation, during childhood may permanently alter brain function.

## Conclusion

In sum, we found accumulation of adverse childhood experiences to increase the risk of psychotropic medication in young adults. Parental educational level was of less importance when adjusting for adverse childhood experiences. The higher risk for future mental health problems among children from lower socioeconomic groups, compared to peers from more advantaged backgrounds, seems to be linked to a higher rate of exposure to adverse childhood experiences.

## Supporting Information

Table S1 Exposure to adverse childhood experiences and risk of psychotropic medication 2006–2008 (OR, 95% confidence intervals).
(DOCX)
