# Supplementary material for: Multi-Exposure and Clustering of Adverse Childhood Experiences, Socioeconomic Differences and Psychotropic Medication in Young Adults
Source: PLoS One. 2013 Jan 16;8(1):e53551. doi: 10.1371/journal.pone.0053551 (PMC3547022; doi:10.1371/journal.pone.0053551)
Supplement: Table S1 — Exposure to adverse childhood experiences and risk of psychotropic medication 2006–2008 (OR, 95% confidence intervals). (DOCX) [file pone.0053551.s001.docx]

*Table S1. Exposure to adverse childhood experiences and risk of psychotropic medication 2006-2008 (OR, 95% confidence intervals)*

| **Adverse childhood experience** | **Women** | **Men** |
| --- | --- | --- |
| Severe crime parents | 1.7 (1.6-1.8) | 2.2 (2.0-2.3) |
| Parental alcohol and/or drug abuser | 1.8 (1.7-1.8) | 2.0 (1.9-2.2) |
| Parental separation and/or single household | 1.6 (1.6-1.7) | 1.8 (1.8-1.9) |
| Household receiving social assistance | 1.8 (1.7-1.9) | 2.1 (2.0-2.2) |
| Child welfare intervention before the age of 12 | 2.3 (2.2-2.5) | 3.1 (2.9-3.4) |
| Mentally ill or suicidal parent | 1.9 (1.8-2.0) | 2.3 (2.2-2.4) |
| Familial death | 1.3 (1.2-1.3) | 1.4 (1.3-1.5) |
| Two or more changes in place of residence | 1.8 (1.6-1.9) | 2.0 (1.9-2.2) |

Adjusted for age, and place of residence
